# Plasma protein profiling reveals dynamic immunomodulatory changes in multiple sclerosis patients during pregnancy

**DOI:** 10.3389/fimmu.2022.930947

**Published:** 2022-07-29

**Authors:** Georgia Papapavlou Lingehed, Sandra Hellberg, Jesse Huang, Mohsen Khademi, Ingrid Kockum, Hanna Carlsson, Ivar Tjernberg, Maria Svenvik, Jonas Lind, Marie Blomberg, Magnus Vrethem, Johan Mellergård, Mika Gustafsson, Maria C. Jenmalm, Tomas Olsson, Jan Ernerudh

**Affiliations:** ^1^ Division of Inflammation and Infection, Department of Biomedical and Clinical Sciences, Linköping University, Linköping, Sweden; ^2^ Division of Bioinformatics, Department of Physics, Chemistry and Biology, Linköping University, Linköping, Sweden; ^3^ Neuroimmunology Unit, Department of Clinical Neuroscience, Center for Molecular Medicine, Karolinska University Hospital, Karolinska Institute, Stockholm, Sweden; ^4^ Department of Clinical Chemistry and Transfusion Medicine, Region Kalmar County, and Department of Biomedical and Clinical Sciences, Division of Inflammation and Infection, Linköping University, Linköping, Sweden; ^5^ Department of Obstetrics and Gynecology, Region Kalmar County, Kalmar, Sweden and Department of Biomedical and Clinical Sciences, Linköping University, Linköping, Sweden; ^6^ Section of Neurology, Department of Internal Medicine, County Hospital Ryhov, Jönköping, Sweden and Department of Biomedical and Clinical Sciences, Linköping University, Linköping, Sweden; ^7^ Department of Obstetrics and Gynecology in Linköping, and Department of Biomedical and Clinical Sciences, Linköping University, Linköping, Sweden; ^8^ Department of Neurology, and Department of Biomedical and Clinical Sciences, Linköping University, Linköping, Sweden; ^9^ Department of Clinical Immunology and Transfusion Medicine, and Department of Biomedical and Clinical Sciences, Linköping University, Linköping, Sweden

**Keywords:** multiple sclerosis, pregnancy, Olink proteomics, plasma, inflammation, cytokines, hormones

## Abstract

Multiple sclerosis (MS) is a chronic autoimmune neuroinflammatory and neurodegenerative disorder of the central nervous system. Pregnancy represents a natural modulation of the disease course, where the relapse rate decreases, especially in the 3^rd^ trimester, followed by a transient exacerbation after delivery. Although the exact mechanisms behind the pregnancy-induced modulation are yet to be deciphered, it is likely that the immune tolerance established during pregnancy is involved. In this study, we used the highly sensitive and specific proximity extension assay technology to perform protein profiling analysis of 92 inflammation-related proteins in MS patients (n=15) and healthy controls (n=10), longitudinally sampled before, during, and after pregnancy. Differential expression analysis was performed using linear models and p-values were adjusted for false discovery rate due to multiple comparisons. Our findings reveal gradual dynamic changes in plasma proteins that are most prominent during the 3^rd^ trimester while reverting post-partum. Thus, this pattern reflects the disease activity of MS during pregnancy. Among the differentially expressed proteins in pregnancy, several proteins with known immunoregulatory properties were upregulated, such as PD-L1, LIF-R, TGF-β1, and CCL28. On the other hand, inflammatory chemokines such as CCL8, CCL13, and CXCL5, as well as members of the tumor necrosis factor family, TRANCE and TWEAK, were downregulated. Further in-depth studies will reveal if these proteins can serve as biomarkers in MS and whether they are mechanistically involved in the disease amelioration and worsening. A deeper understanding of the mechanisms involved may identify new treatment strategies mimicking the pregnancy milieu.

## Introduction

Multiple sclerosis (MS) is a chronic autoimmune inflammatory and degenerative disorder of the central nervous system (CNS) that leads to demyelination and axonal loss (1). Its high degree of heterogeneity regarding clinical and pathological manifestations remains challenging and an increased understanding of central disease-promoting and alleviating mechanisms is needed to develop more effective treatments and biomarkers for individualized treatment (2–4). Indeed, despite the growing number of disease-modifying treatments, many MS patients continue to deteriorate due to insufficient efficacy and inadequate personalized treatment strategies.

A yet unexplored area to improve our knowledge of the underlying disease mechanisms is linked to the favorable effect that pregnancy has on the disease course. More precisely, MS temporarily improves during pregnancy, especially during the third trimester, where the reduction in relapse rate reaches 70% (5, 6). A transient rebound follows delivery, before the disease activity returns to the pre-pregnancy levels. The observed disease amelioration is likely linked to the state of immunological tolerance induced during pregnancy to avoid rejection of the semi-allogeneic fetus (7). This, in combination with endocrine alterations, mainly the increased levels of the pregnancy hormones progesterone (P4) and estrogen, which sharply decline after delivery mimicking the transient improvement and worsening during and after pregnancy, respectively, highlight a potential role for immune-endocrine interactions. A better understanding of how pregnancy can alter the disease activity in MS could provide important insights into the disease pathogenesis and open up possibilities for new treatment strategies mimicking the milieu during pregnancy.

The involvement of the peripheral immune system in driving MS (1, 8), together with the pronounced effect of pregnancy on the disease course (5, 6), strongly suggest that systemic changes are relevant for explaining the transient improvement and worsening during pregnancy. The frequency of immune cells involved in MS appears to be largely unaltered during pregnancy in patients with MS (9), suggesting that functional differences rather than changes in cell proportions may underlie disease improvement. Although some studies have investigated systemic immune modulation in MS during pregnancy from various cellular and molecular angles (9–14), extensive profiling of inflammatory proteins has not been performed. Furthermore, the low abundance in plasma of most inflammatory proteins has hindered a thorough mapping of systemic protein changes in MS. However, nowadays, the recent development of high sensitivity proteomic technologies allows for the detection of low abundant protein changes in the circulation (15, 16). One such methodology, the proximity extension assay (PEA) (17), has successfully revealed potential biomarker candidates in plasma in MS (18). Indeed, plasma proteins would constitute optimal biomarkers in MS as well as in other inflammatory diseases. We here report a protein profiling analysis in MS and healthy controls (HC), using the highly sensitive and yet highly specific PEA technology to decipher the dynamic changes of protein levels during pregnancy, with the goal to identify protein targets that are altered in pregnancy and could potentially in amelioration or perturbation of the disease. Our findings demonstrate that the dynamic changes of proteins follow the same pattern as the clinical activity of MS*, i.e.*, the most pronounced changes are found in the third trimester, coinciding with the most pronounced improvement, and they reverse post-partum. The findings highlight both well-known and less considered proteins as being mechanistically relevant and potential biomarkers for disease amelioration and exacerbation.

## Materials and methods

### Study cohort

Plasma samples were obtained from pregnant women with relapsing-remitting MS (n=15) and pregnant HC (n=10). The women were followed longitudinally with repeated blood sampling during pregnancy; 1^st^ trimester [MS; gestational week (gw) median (range) 9.6 (5.1-9.6), HC; gw 11.5 (9.9-13.6) 2^nd^ trimester [MS; gw 25.0 (23.1-26.7), HC; gw 25.3 (24.3-28.0)], 3^rd^ trimester [MS; gw 35.1 (33.0-36.1), HC; gw 35.3 (34.3-37.3)] and, in addition, post-partum [MS; week 6.0 (5.0-7.0), HC; week 8.6 (5.0-12.7)]. Sampling time at 1^st^ trimester and post-partum differed significantly between the groups, where in both cases, the sampling in women with MS was performed earlier than in HC. MS patients were recruited at Karolinska University Hospital, Solna, Sweden (n=10), at Linköping University Hospital, Linköping, Sweden (n=4) and at Ryhov County Hospital, Jönköping, Sweden (n=1). All HC donors were recruited at the maternity clinic in Region Kalmar County, Sweden. For 13 of the MS patients, a baseline sample (pre-pregnancy) was collected at a consultation meeting with their physician related to treatment options prior to a desired pregnancy. The remaining two MS patients were included during early pregnancy. All pregnant HC were informed about the study at their first routine antenatal visit at the maternity clinic. To obtain an equivalent to the pre-pregnancy sample in MS for the healthy group, an independent group of non-pregnant healthy female blood donors (n= 15) was recruited at Linköping University Hospital, Linköping, Sweden. Blood samples were collected in EDTA tubes (10 ml, BD Vacutainer, Beckton Dickinson, New Jersey, USA) using identical protocols and procedures at the four centers. Samples were centrifuged at 1500xg for 15 min at room temperature, within 2 hours from sampling, and the plasma was frozen at -70°C until analysis. At every visit, the healthy pregnant women filled in a health survey form, whereas the MS patients answered a questionnaire that was filled in by a research nurse. Disability was assessed for the MS patients at inclusion as well as post-partum by a neurologist using the expanded disability status scale (EDSS) (19).

**Figure 1:**
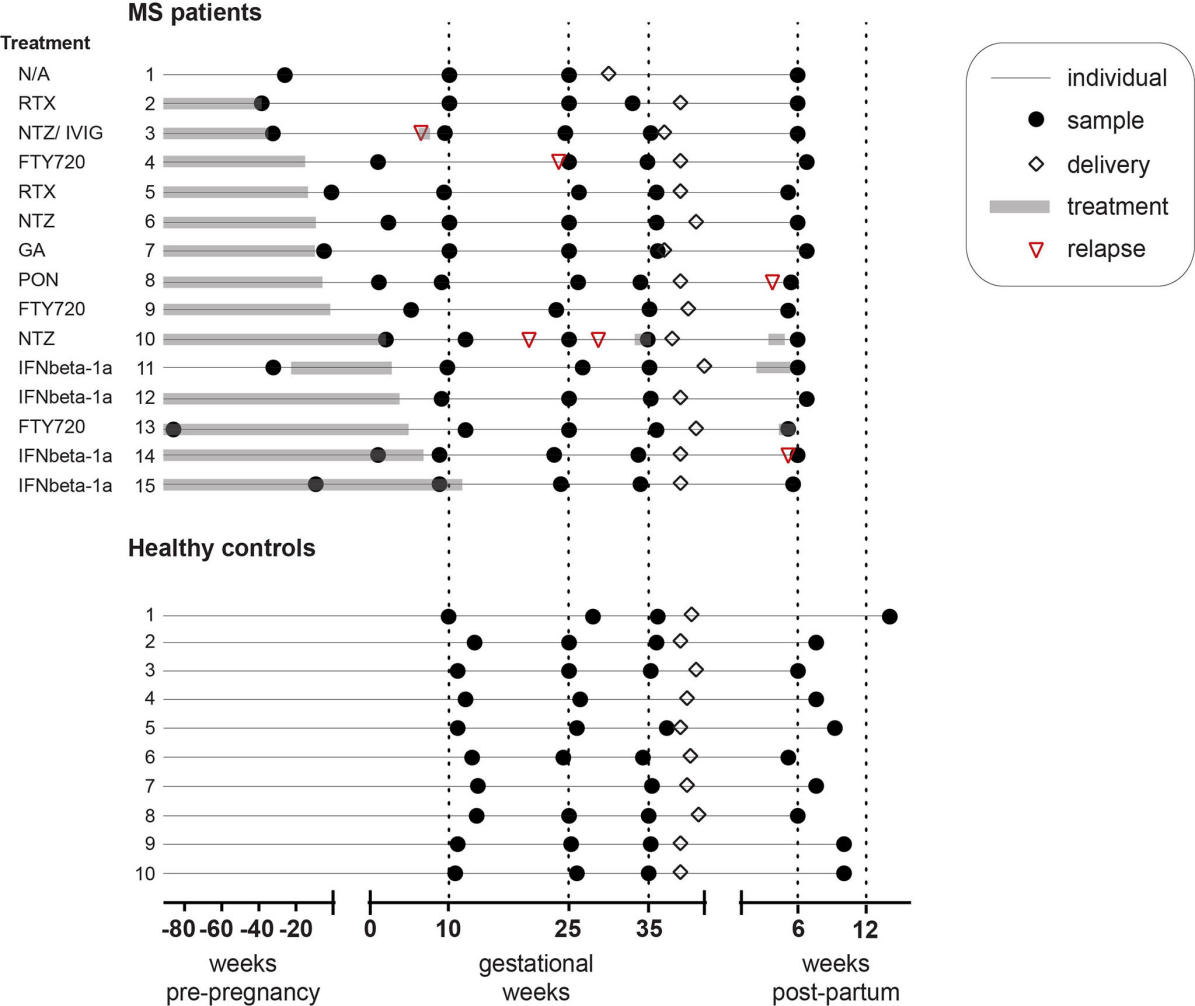
Study cohort characteristics. Schematic representation of the cohort consisting of pregnant MS patients (n = 15) and healthy controls (n = 10). Shown is the exact sampling time in weeks (black circles) for the pre-pregnancy sample (only in the MS group), as well as in the 1^st^, 2^nd^, and 3^rd^ trimesters of pregnancy and post-partum. Delivery (white rhombus), treatment duration (grey rectangle) and relapse (red triangle) are also depicted. MS patients were recruited at Karolinska University Hospital, Solna, Sweden (n = 10), at Linköping University Hospital, Linköping, Sweden (n = 4) and at Ryhov County Hospital, Jönköping, Sweden (n = 1). All healthy pregnant donors (n = 10) were recruited at Kalmar County Hospital, Kalmar, Sweden. MS patient 3 had immune globulin intravenous at gestational week 6.4, patient 10 had Natalizumab at gestational week 34.3 and week 3.7 post-partum, patient 11 had IFNbeta-1a at week 2.4-5.4 post-partum, and patient 13 had Fingolimod at week 4.9 post-partum. FTY720, Fingolimod (n = 3); GA, Glatiramer acetate (n = 1); IFNbeta-1a, Interferon beta-1a (n = 4); IVIG, Intravenous immune globulin; MS, multiple sclerosis; NTZ, Natalizumab (n = 3); PON, Ponesimod (n = 1); RTX, Rituximab (n = 2); N/A, Not applicable (n = 1).

Eligible for the study were women of Caucasian ethnicity, aged 18-45 years, who were generally healthy and apart from MS (in the MS group) did not have any other immune-associated or other severe diseases and were not on immunomodulatory treatments other than related to MS. Women with assisted pregnancy or a history of previous obstetric complications were not eligible for inclusion in the study. All pregnancies were singleton. No statistical difference was noted in age or body mass index between the groups. However, the incidence of miscarriages was higher in MS than in HC (p=0.04). In the HC group, there were no reported major pregnancy complications except for one case of severe preeclampsia in gw 38. In the MS group, one case of preterm birth (week 31) was noted, and in addition, one case of hypothyroidism, one case of asthma, and one case of polycystic ovarian syndrome. The characteristics of the participants are summarized in **Table 1** and **Figure 1**. The study was performed in accordance with the Helsinki Declaration ethical principles for medical research and was approved by the Regional ethical review board in Linköping (2012/402-31). All participants signed an informed consent.

**Table 1:** Study cohort characteristics.

Variable	MS patients (n = 15)	Healthy pregnant controls (n = 10)	Healthy non-pregnant (n = 15)
Pre-pregnancy, n	13	N/A	N/A
1st trimester, n	14	10	N/A
2nd trimester, n	15	9	N/A
3rd trimester, n	14	10	N/A
Post-partum, n	15	10	N/A
Delivery (weeks), mean ± SD	39.0 ± 2.6	39.9 ± 0.9	N/A
Age (years), mean ± SD	31.6 ± 3.7	29.1 ± 4.8	30.5 ± 6.0
BMI, mean ± SD	24.1 ± 2.9	22.7 ± 7.3	Not available
Gravidity, mean ± SD	1.1 ± 1.3	0.3 ± 0.7	N/A
Previous miscarriages, mean ± SD	0.5 ± 1.1	0	N/A
Previous live births, mean ± SD	0.3 ± 0.6	0.3 ± 0.7	N/A
Fetal sex	8 males/7 females	3 males/7 females	N/A
Mode of delivery			
Vaginal delivery, n	11	9	N/A
Caesarean section, n	4	1	N/A
Disease duration, mean ± SD	6.0 ± 5.4^a^	N/A	N/A
Disease severity			
EDSS at inclusion, median (range)	1.0 (0 -5)^b^	N/A	N/A
EDSS post-partum, median (range)	1.0 (0 -5)^c^	N/A	N/A
Treatment wash-out (weeks)			
from pre-P sampling, median (range)	2.4 (0 - 16)	N/A	N/A
from 1^st^ trimester sampling, median (range)	10 (0 - 48)	N/A	N/A

Cohort characteristics of multiple sclerosis patients (MS) and healthy controls. ^a^disease duration was missing for one individual, data on EDSS was missing for ^b^1 individual and ^c^5 individuals. BMI, Body mass index kg/m^2^; EDSS, Expanded disability status score, pre-P, pre-pregnancy; SD, standard deviation.

### Protein profiling of plasma proteins

A total of 92 proteins were measured in the plasma samples with PEA technology using the Olink INFLAMMATION panel in 2017 (**Table S1**). The panel consists of pre-selected proteins which are mainly involved in inflammation and immune response/activation but can also be implicated in other cellular functions such as apoptosis, oxidative stress, and development. Briefly, the method includes the incubation of 1 μl sample with a mixture of oligonucleotide-labeled antibody probe pairs, each pair specific for one protein in the panel. When the two probes are in close proximity through binding to different epitopes on the target protein, their complementary sequences form a polymerase chain reaction (PCR) target by a proximity-dependent DNA polymerization event, which is detected and quantified using quantitative PCR (17). Protein levels are expressed on a relative log2 scale with arbitrary units presented as normalized protein eXpression (NPX). One sample in the non-pregnant control group failed the quality control due to high variability and was removed from further analyses. Limit of detection (LOD) was determined for each protein and proteins below the LOD were assigned the LOD value. Proteins detected in less than 50% of the samples were excluded from further analyses, which resulted in 20 proteins (22%) being removed. Details on the call rate and LOD values for all proteins are listed in **Table S1**. Raw data with the NPX values for all proteins is shown in **Table S2**.

Since we know that sample handling can impact the levels of some proteins (20) and since our MS and HC groups were collected at different sites, we evaluated if sample handling time could affect our measured proteins despite that all samples were handled within 2 hours. When correlating levels of proteins known to be markers of sample handling (AXIN-1, STAMBP, ST1A1, CASP-8 and SIRT2) with the actual handling time of each sample, no correlations were found (data not shown).

### Measurement of hormones in plasma

Pregnancy hormones P4 and estradiol (E2) were measured in all plasma samples by electrochemiluminescence technology according to the manufacturer’s instructions (Roche Diagnostics Scandinavia AB, Sweden) at the Laboratory of Clinical Chemistry at Linköping University Hospital, Linköping Sweden. P4 levels were expressed in nmol/l and E2 in pmol/l. The hormone levels for the individual samples are listed at the top of **Table S2**.

### Statistical analysis

For the calculation of the z scores, the pre-pregnancy sample was used [example for 3^rd^ trimester: (mean _3rd trim_ –mean _pre-P_)/std _pre-P_)]. Differential expression analysis was performed in R (Version 4.1.3; Boston, MA, USA) using linear modelling available through the R package limma (21), which has been shown to be powerful in detecting differences in protein abundance (22–24). The *duplicateCorrelation* function was used with time-point as a covariate and patient ID as a random effect. For the differentially expressed proteins (DEPs), an adjusted p-value<0.05 (Benjamini-Hochberg) was considered statistically significant, unless stated that nominally significant proteins are shown (p-value<0.05). For the comparison between MS and HC, all values were corrected for the 1^st^ trimester time-point in the limma model. Odds ratios and p-values for overlapping proteins in the Venn diagrams were calculated in R using *fisher.test* for Fisher’s exact test. Pearson correlations between hormones and protein levels were performed using the cor.test function in R and r values > 0.5 in absolute value and a p-value<0.05 were considered significant. For the comparison of the demographic and clinical parameters between the groups, one-way ANOVA was used for comparing 3 groups, unpaired t-test for 2 groups, and Fisher’s exact test for categorical data. Graphs were made in GraphPad Prism (Version 8.0; San Diego, CA, USA) and Venn diagrams and volcano plots in R. The R-code used in all analyses is publicly available in the github repository at https://github.com/sanhe374/MS_Pregnancy_proteomics.

## Results

### Dynamic changes of inflammation-related proteins during pregnancy

**Figure 2:**
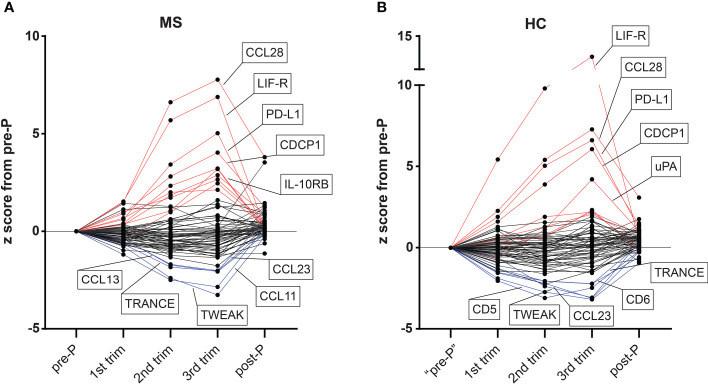
Overview of dynamic changes in plasma proteins during pregnancy in MS and HC. **(A, B)** The levels of all 72 detectable proteins (detected in >50% of samples) measured by the proximity extension assay methodology in plasma samples from MS patients (n=15) and healthy controls (HC, n=10) are presented as z scores from the pre-pregnancy sample (example for 3^rd^ trimester: (mean _3rd trim_ –mean _pre-P_)/std _pre-P_). For the pre-pregnancy sample in the HC group, an independent non-pregnant group was included (n = 14, after exclusion of one sample due to failure in quality control). Proteins with ≥ +2 z score in the 3^rd^ trimester *versus* pre-pregnancy are colored in red and those with ≥ -2 z score are shown in blue. The top five up and top five downregulated proteins in each group are highlighted in the figure. HC, healthy controls; MS, multiple sclerosis; post-P, post-partum; pre-P, pre-pregnancy; trim, trimester.

To investigate changes in plasma proteins during pregnancy in MS patients (n=15) and HC (n=10), we analyzed 92 inflammation-related proteins using the highly sensitive proteomic immunoassay PEA (17). To get an initial and general understanding of the dynamics of protein changes during pregnancy, we plotted the relative protein changes in each trimester based on the z score from the pre-pregnancy sample. In both MS and HC, a substantial number of proteins changed gradually throughout pregnancy and reversed post-partum (**Figure 2**). Among the proteins that increased the most (highest z score) in the 3^rd^ trimester compared to pre-pregnancy in both MS and HC were CCL28, LIF-R, PD-L1, and CDCP1, while the common proteins with the highest negative z score in absolute value were TWEAK, TRANCE and CCL23 (**Figure 2A, B**). Overall, many proteins displayed a dynamic pattern of changes throughout pregnancy, with the most prominent increase or decrease taking place in the 3^rd^ trimester and reversing after delivery.

### Differentially expressed proteins during pregnancy in MS

To gain precise insight into the changes that occur throughout pregnancy in MS patients, we performed linear modelling analysis to identify DEPs. The least number of DEPs was observed in the comparisons of 1^st^ trimester *versus* pre-P (n=13 DEPs, false discovery rate (FDR)<0.05), 3^rd^ *versus* 2^nd^ trimester (n=11 DEPs), and post-P *versus* pre-P (n=12 DEPs), indicating that these time-points were relatively similar to each other (**Table S3**). In contrast, 33 out of a total of 72 proteins (46%) were differentially expressed in the 3^rd^ *versus* 1^st^ trimester (FDR<0.05) and 44 proteins (61%) were differentially expressed post-partum *versus* 3^rd^ trimester. Notably, this pattern follows the dynamics of clinical activity in MS, with the most prominent improvement occurring during the 3^rd^ trimester of pregnancy, followed by a worsening post-partum (5). We, therefore, decided to focus our further analysis on these two time-points.

**Figure 3:**
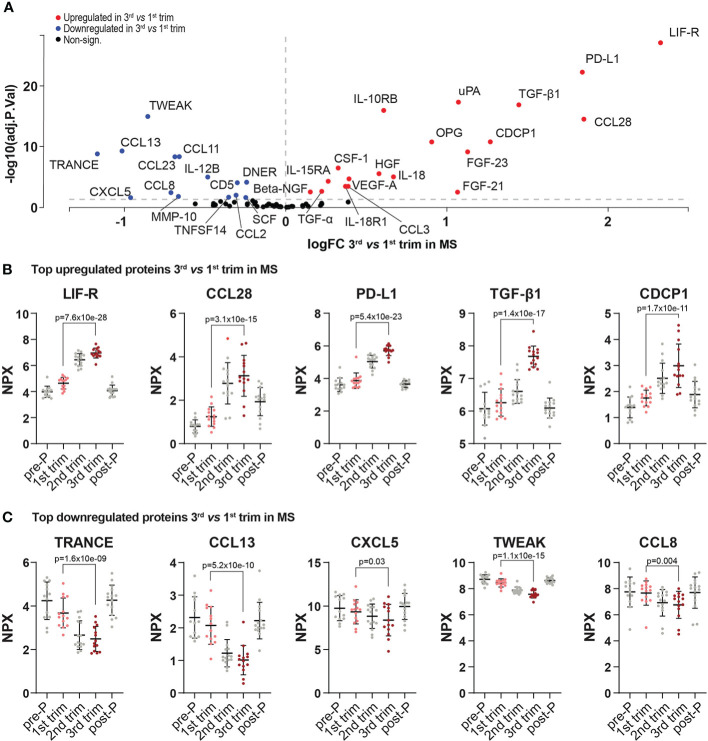
Differentially expressed proteins in 3^rd^ trimester *versus* 1^st^ in MS. **(A)** Volcano plot of the differentially expressed proteins (DEPs) at 3^rd^ trimester *versus* 1^st^ in MS patients. DEPs were determined using linear models and Benjamini-Hochberg correction for multiple comparisons (FDR<0.05). Up-and downregulated DEPs are shown as red and blue, respectively. Non-significant proteins are shown in black. **(B, C)** Dot plots of the top five up-and downregulated proteins in the 3^rd^ trimester *versus* 1^st^ (based on logFC, FDR<0.05). To visualize the entire dynamic pattern, distribution is shown at all time-points (pre-pregnancy, during the three trimesters of pregnancy and post-partum), but only 1^st^ and 3^rd^ trimesters are shown in color and with the respective adjusted p-value. Protein levels are presented as NPX values on a log2 scale. All included proteins were also differentially expressed between post-partum and 3^rd^ trimester. Mean ± standard deviation is shown. logFC, logFoldChange; MS, multiple sclerosis; NPX, normalized protein eXpression; pre-P, pre-pregnancy; post-P, post-partum; trim, trimester.

The changes between 3^rd^ and 1^st^ trimesters consisted of 19 upregulated and 14 downregulated proteins (FDR<0.05, **Figure 3A–C**, **Figures S1A, B** and **Table S3**). Several known anti-inflammatory proteins including for example LIF-R, PD-L1, IL-10RB and TGF-β1 were upregulated in pregnancy and so did the chemokine CCL28 (**Figure 3B** and **Figure S1A**). Conversely, amongst the downregulated proteins were several pro-inflammatory mediators such as IL-12B, the chemokines CCL8, CCL13 and CXCL5, as well as the tumor necrosis factor family members TRANCE and TWEAK (**Figure 3C** and **Figure S1B**).

**Figure 4:**
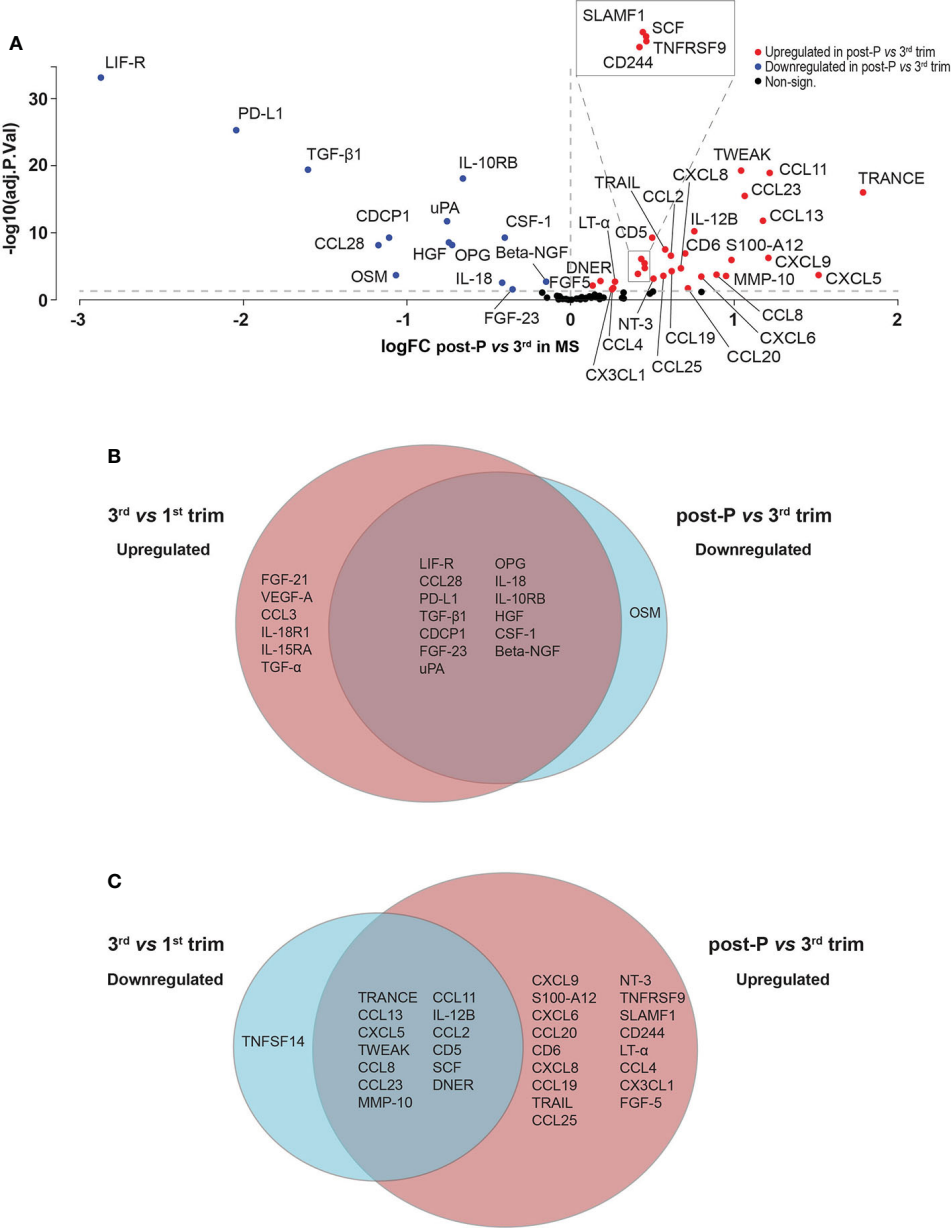
Differentially expressed proteins in post-partum *versus* 3^rd^ trimester in MS. **(A)** Volcano plot of the differentially expressed proteins (DEPs) at post-partum *versus* 3^rd^ trimester in MS patients (n = 15). DEPs were determined using linear models and Benjamini-Hochberg correction for multiple comparisons (FDR < 0.05). Up-and downregulated DEPs are shown as red and blue, respectively. Non-significant proteins are shown in black. **(B, C)** Venn diagrams depicting the common and unique DEPs comparing up- and downregulated DEPs in 3^rd^ *versus* 1^st^ trimester and post-partum *versus* 3^rd^. logFC, logFoldChange; MS, multiple sclerosis; post-P, post-partum; trim, trimester.

Out of the 44 proteins that were differentially expressed post-partum compared to the 3^rd^ trimester, 30 were upregulated and 14 were downregulated (FDR<0.05, **Figure 4A**, **Figure S1A, B** and **Table S3**). We hypothesized that many of the induced changes during pregnancy would reverse post-partum, mirroring the temporary improvement and worsening of the disease. Indeed, there was a highly significant overlap between the proteins that increased during the 3^rd^ trimester and decreased post-partum (13/33 proteins, odds ratio (OR): 98.9, p=4.85e-09, Fisher’s exact test, **Figure 4B**) and, conversely, between the downregulated proteins during the 3^rd^ trimester and the ones upregulated post-partum (13/44 proteins, OR: 29.9 , p=1.73e-05, **Figure 4C**). These overlaps further support the hypothesis of a reversal of protein levels after pregnancy.

To evaluate if the results were affected by treatment in the MS group, we performed the same differential expression analysis removing 6 individuals (numbers 5, 9, 10, 13, 14 and 15 in **Figure 1**) who had their 1^st^ trimester samples potentially affected by treatment. Although the sample size was reduced, we observed the same DEPs when comparing 3^rd^ *versus* 1^st^ trimester and post-partum *versus* 3^rd^ trimester, with the top upregulated and downregulated proteins remaining unchanged (data not shown). The only exception was CXCL5 which was only nominally significantly downregulated in the 3^rd^ *versus* 1^st^ trimester comparison and did not survive FDR correction.

### Differentially expressed proteins during pregnancy in HC

**Figure 5:**
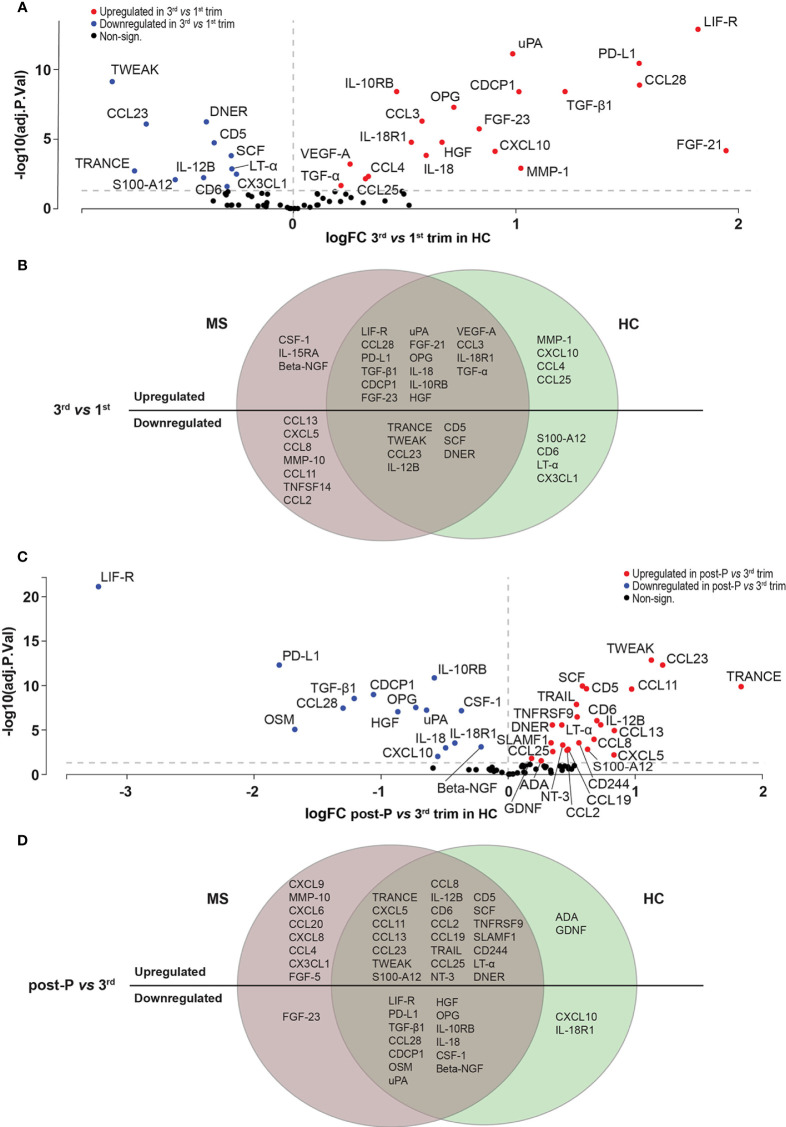
Differentially expressed proteins in HC. **(A)** Volcano plot showing the differentially expressed proteins (DEPs) at 3^rd^ trimester *versus* 1^st^ in healthy controls (HC, n = 10). DEPs were determined using linear models and Benjamini-Hochberg correction for multiple comparisons (FDR<0.05). Up-and downregulated DEPs are shown as red and blue, respectively. Non-significant proteins are shown in black. **(B)** Venn diagram of the overlapping and unique up-and downregulated DEPs between MS (n=15) and HC (n=10) at 3^rd^ trimester *versus* 1^st^. **(C)** Volcano plot showing the differentially expressed proteins (DEPs) post-P compared to 3^rd^ trimester in HC. **(D)** Venn diagram of the common and unique up- and downregulated DEPs between MS (n=15) and HC (n = 10) at post-partum *versus* 3^rd^ trimester. HC, healthy controls; logFC, logFoldChange; MS, multiple sclerosis; post-P, post-partum; trim, trimester.

Although the equivalent to the pre-pregnancy sample in the healthy group (obtained from a non-pregnant group) showed, similar to the MS group, moderate differences compared with the 1^st^ trimester (n=11 DEPs, data not shown), we decided to exclude it from further analyses since it was not paired to the remaining pregnancy and post-pregnancy samples. Consistent with the findings in MS, the least number of DEPs in HC was also noted in the comparison between 3^rd^ and 2^nd^ trimesters (n=20 DEPs, FDR<0.05) followed by the 2^nd^ *versus* 1^st^ trimester comparison (n=24 DEPs, **Table S4**). In contrast, and in line with the pattern in MS, 31 out of 72 (43%) proteins were differentially expressed in the 3^rd^ *versus* 1^st^ trimester (FDR<0.05), and 39 proteins (54%) were differentially expressed post-partum *versus* 3^rd^ trimester. Out of the 31 DEPs in the 3^rd^ trimester, 20 were upregulated and 11 were downregulated (FDR<0.05, **Figure 5A**, **Figure S2A, B** and **Table S4**). A substantial number of these proteins showed a significant overlap with the proteins changing in MS patients (16/20 upregulated proteins, OR: 57.7, p=9.22e-10, and 7/11 downregulated proteins, OR: 12.7, p=5.14e-4, **Figure 5B**). When comparing post-partum to 3^rd^ trimester, 24 of the 39 DEPs were upregulated and 15 were downregulated (FDR<0.05, **Figure 5C** and **Figure S2A, B** and **Table S4**). Also here, there was a highly significant overlap between MS and HC (22/54 upregulated proteins, OR: 50.4, p=6.45e-10, and 13/29 downregulated proteins, OR: 192.4, p=7.44e-10, **Figure 5D**).

When comparing the proteins that were upregulated in the 3^rd^ *versus* 1^st^ trimester to the ones downregulated post-partum *versus* 3^rd^ trimester, there was a highly significant overlap (12/35 proteins, OR: 22.8, p=2.5e-06, **Figure S3A**), showing, in accordance with the findings in MS, that many of the proteins that increase in the 3^rd^ trimester reverse post-partum. Similarly, there was a highly significant overlap between the downregulated proteins in the 3^rd^ trimester and the ones upregulated post-partum (10/35 proteins, OR: 31.7, p=3.20e-05, **Figure S3B**). Taken together, the HC show a similar pattern of dynamic changes during pregnancy and share a lot of commonly regulated proteins with the MS patients.

The overall similarities between MS and HC were further supported by principal component analysis (PCA), where samples did not cluster based on group within each time-point (**Figure S4A, B**). We, therefore, decided to combine the two groups at each time-point to increase the statistical power to detect DEPs. Combining the groups did not change the results (data not shown), indicating that the observed changes are representative and not influenced by increasing sample size.

### MS and HC do not depict substantial differences during pregnancy

**Figure 6:**
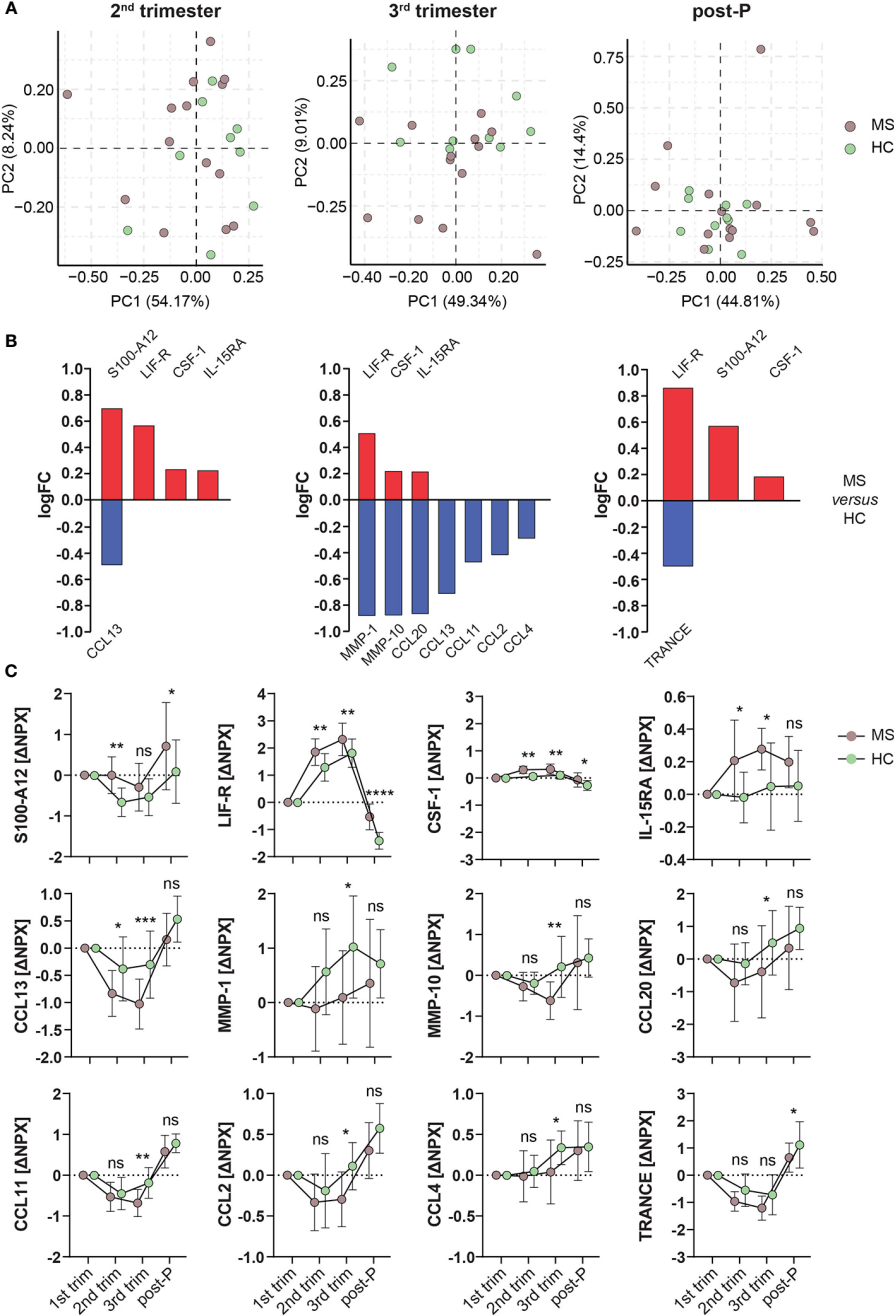
Differentially expressed proteins in MS *versus* HC. **(A)** Principal component analysis (PCA) plots of protein changes in MS patients and healthy controls (HC) in the different trimesters. PCA was performed on the ΔNPX values, which were computed by subtraction of the value for the 1^st^ trimester Brown dots represent MS patients (n = 14, individual 4 in the MS group is excluded due to missing 1st trimester sample) and green dots represent HC (n = 10). **(B)** Bar graphs showing the nominally differentially expressed proteins (DEPs, p < 0.05) using linear models in MS *versus* HC. Proteins higher in MS *versus* HC are shown in red, and the ones lower in MS are shown in blue. **(C)** Plots of the ΔNPX values of the nominally DEPs shown in **(B)** which are higher or lower in MS (n = 14) compared to HC (n = 10). From these proteins, CCL11 and CCL13 in the 3^rd^ trimester and LIF-R post-partum survived FDR correction. Mean ± standard deviation is shown. *p < 0.05, **p < 0.01, ***p < 0.001, ****p < 0.0001, ns; not significant. HC, healthy controls; logFC, logFoldChange; MS, multiple sclerosis; NPX, normalized protein eXpression; PC, principal component; post-P, post-partum; trim, trimester.

Based on the observed similarities between MS and HC, we went further to investigate any potential differences by directly comparing MS and HC. To remove potential effects from differences in sample site collection between these groups, all comparisons were made correcting for the 1^st^ trimester time-point. Initial unsupervised clustering of the samples using PCA showed a high degree of similarity between MS and HC during pregnancy and post-partum (**Figure 6A**) and accordingly, only 3 out of the 12 nominally differentially expressed (p<0.05) proteins between MS and HC (CCL11 and CCL13 in the 3^rd^ trimester and LIF-R post-partum) survived FDR correction. From these 12 proteins, LIF-R and CSF-1 were higher in MS compared to HC at all three time-points, while S100-A12 was higher in the 2^nd^ trimester and post-partum and IL-15RA in the 2^nd^ and 3^rd^ trimesters (**Figure 6B**). Interestingly, out of these proteins, only LIF-R was shared between MS and HC, being upregulated in the 3^rd^ trimester *versus* 1^st^, while CSF-1 and IL-15RA were increased in MS only and S100-A12 was downregulated in HC only (**Figure 5B**). The nominally significant proteins that were lower in MS compared to HC included CCL13 in the 2^nd^ trimester and TRANCE post-partum. Additionally, seven proteins including the metalloproteinases MMP-1 and MMP-10 as well the chemokines CCL2, CCL4, CCL11, CCL13, and CCL20 were lower in MS compared to HC in the 3^rd^ trimester (**Figure 6B**). The ΔNPX values for all nominally significant proteins comparing the two groups are shown in **Figure 6C**.

### Correlations with hormones

Since the concentrations of the pregnancy hormones P4 and E2 follow the observed changes in disease activity in MS patients during pregnancy, we measured the levels of P4 and E2 in all plasma samples from all time-points. As expected, during normal pregnancy, the concentrations of the hormones rise gradually throughout pregnancy, peaking in the 3^rd^ trimester, and decline post-partum (**Figures S5A**, **S6A**). There was no difference in the hormone levels between MS and HC (data not shown), showing that the hormonal changes associated with pregnancy occur equally. Correlation analysis of the hormone levels with the proteins revealed that many DEPs (n= 16 for P4, n=14 for E2) correlated with the hormone levels during pregnancy (**Figures S5B**, **Figure S6B**).

## Discussion

The profound effect and dynamics of the pregnancy-associated disease activity on MS underscore the potential to identify immune-modulatory mechanisms and protein markers that correlate with improvement and worsening of the disease. We here report the first large-scale immune-related plasma protein profiling of longitudinal paired samples in MS patients during and after pregnancy. Taken together, our data demonstrates the dynamic changes in inflammation-related proteins that take place during pregnancy. The changes were found to be similar in MS patients and HC, underlining the general impact of the immunological regulation during pregnancy. The alterations are most pronounced in the 3^rd^ trimester of pregnancy and reverse after delivery, following the same pattern as the changes in the clinical activity of MS, *i.e.*, amelioration during pregnancy, especially in the 3^rd^ trimester, and exacerbation post-partum. In addition, the dynamic protein changes are in line with the known increased susceptibility and severity of some infections, especially in the 3^rd^ trimester, which are linked to the immune adaptations taking place in pregnancy (25). The study highlights potential mechanisms and biomarker candidates such as PD-L1, LIF-R, TGF-β1, and CCL28 related to amelioration and TRANCE, TWEAK, CCL8, CCL13, and CXCL5 related to disease worsening.

It has recently become evident that the immune alterations during normal pregnancy are precisely timed and can even predict gestational age and time of delivery (26), underlining the importance of immune regulation for a successful pregnancy. Overall, our findings of protein dynamics during pregnancy were mostly shared between MS patients and HC, showing that MS patients undergo the same changes as healthy pregnant women. Indeed, pregnancy is a huge challenge for the maternal immune system, and therefore the finding of similar regulation in MS and HC is not surprising. Still, this immune regulation benefits both groups in terms of fetal tolerance, and additionally, patients with MS in general benefit with a temporary improvement. Recently, large studies have investigated protein changes in normal pregnancies (27–29). Out of studies using the same PEA methodology, the one by Hedman *et al.*, although including different time-points of sampling than we did, reported protein changes during the 2^nd^ trimester of pregnancy that mostly had the same direction as in our study (28). Furthermore, our comparisons between the 3^rd^ trimester and post-partum revealed findings that are well in accordance with the study by Bränn *et al.*, where 290 women were sampled during late pregnancy and early post-partum. The proteins reported being higher in late pregnancy compared to post-partum included LIF-R, TGF-β1, CCL28, OSM, and FGF-21, while post-partum TRANCE, TWEAK, and CCL11 increased the most compared to pregnancy (27). In addition to these proteins, we found that PD-L1 consistently ranked among the proteins with the most profound changes. PD-L1 had a low overall detectability and was therefore not included in the analysis by Bränn *et al.* It was, however, found to be detectable in late pregnancy, while in contrast, it was mostly undetectable post-partum (27), which is in line with our finding of higher levels in the 3^rd^ trimester and downregulation post-partum. PD-L1/PD-1 signaling has a well-known pivotal role in maintaining T cell homeostasis and peripheral tolerance by inhibiting T cell activation and promoting differentiation of regulatory T (Treg) cells (30). Of note, an imbalance in PD-L1/PD-1 signaling has been suggested in MS pathogenesis and proposed as a potential target in MS treatment, although the role of soluble PD-L1 is not fully established (30). Interestingly, however, soluble PD-L1 fusion protein was shown to ameliorate experimental autoimmune encephalomyelitis (EAE) (31). Our finding of dynamic regulation during pregnancy further supports the notion of PD-L1 as a central immune-modulatory protein of potential use as a biomarker or treatment target in MS.

In addition to PD-L1, several other well-known anti-inflammatory proteins such as LIF-R, TGF-β1, IL-10RB, and CSF-1 increased in the 3^rd^ trimester of pregnancy. LIF-R is the receptor for the cytokines LIF and OSM, which belong to the IL-6 cytokine family (32). LIF is of known relevance in both MS and pregnancy. In MS it is believed to have neuroprotective properties since it enhances Treg numbers (33). Moreover, it was found to be produced by myelin-reactive T cells from MS patients and proposed to protect against TNF-induced oligodendrocyte apoptosis (34). Levels of LIF are increased in serum and cerebrospinal fluid in MS (34, 35), and the expression of LIF-R is also increased in circulating immune cells of MS patients (33). In human reproduction, LIF is believed to have an important role in implantation, the establishment of pregnancy and embryo development (36). Although the information is limited, given its potential role in neuroprotection and immune modulation, the LIF/LIF-R pathway is a candidate for further exploration, as supported by our present findings. TGF-β1 is another well-known immune modulator of high relevance in both MS and pregnancy. Here, TGF-β was measured in the form of the LAP TGF-β1, the latency-associated peptide required to maintain the protein TGF-β. Both LAP TGF-β1 and TGF-β have well-known immunosuppressive properties (37, 38). TGF-β is a main inducer of Treg cell development and function, which are key elements in the immune modulation of both MS and pregnancy (39, 40). However, since active TGF-β1 is difficult to measure, it is not optimal as a biomarker, and its broad biological effects complicate its use in treatment (41). IL-10 and CSF-1 are also well-known anti-inflammatory and immune-modulatory proteins. IL-10 inhibits T cell activation and induces Treg cells, and both IL-10- and CSF-1 signaling are involved in the polarization of M2-type macrophages, which are beneficial to counteract inflammatory M1 pathology at the fetal-maternal interface (42, 43) and in the CNS (44). However, whether the circulating levels of IL-10RB and CSF-1 are associated with beneficial effects on CNS inflammation remains to be determined.

Other proteins that were upregulated during the 3^rd^ trimester included CCL28 and CDCP1, which are difficult to classify according to limited knowledge and potentially dual effects. CCL28 has been linked to the recruitment of Treg cells (46, 47), however CCL28 and its receptor CCR10 were also proposed to be pathogenic in rheumatoid arthritis (47). CDCP1 is believed to modulate immune responses upon T cell activation and has been implicated in inflammatory responses and autoimmunity (48). Knockout of the CDCP1 gene in EAE resulted in attenuated disease severity and infiltration of IFN-γ and IL-17-producing T cells (49).

Downregulated proteins during pregnancy included three proteins where single nucleotide polymorphisms have previously been linked to MS susceptibility through genome-wide association studies (50); TNFSF14, which was downregulated in MS only, CD6, which was downregulated in HC only, and IL-12B, which was downregulated in both groups. In addition, CD6 and IL-12B were upregulated post-partum in both MS and HC. These findings suggest that these genes/proteins might reflect disease activity in addition to disease susceptibility. Interestingly, we recently suggested that IL-12B, together with CD5, CCL3, and CXCL9, could serve as MS biomarkers in cerebrospinal fluid (18). Additional proteins downregulated in the 3^rd^ trimester included several inflammatory chemokines, suggesting their potential role in promoting the disease.

OSM and HGF are two additional interesting proteins since, using the same PEA technology, we recently showed that they were upregulated in MS compared to controls in plasma (18). Both proteins are pleiotropic with both beneficial and disease-promoting effects (32, 51). Our findings of HGF being upregulated in the 3^rd^ trimester and downregulated together with OSM post-partum in both MS and HC may support a beneficial role of these proteins.

Among the proteins that increased post-partum compared with the 3^rd^ trimester were two members of the TNF family, TRANCE and TWEAK. Both are multifunctional cytokines involved in many biological processes (52, 53). In the context of neuroinflammation, inactivation of TRANCE in EAE was associated with decreased infiltration of Th17 cells through the blood-brain barrier due to reduced secretion of CCL20 by astrocytes (54). TWEAK has also been implicated in the pathophysiology of inflammatory diseases, including MS (55, 56). Given their potential disease-promoting effects, the increase in TRANCE and TWEAK post-partum could be associated with the parallel increase in disease activity. However, the changes in these proteins are difficult to interpret, given their multiple actions. For example, both TRANCE and TWEAK have been associated with tolerance-inducing properties in the context of pregnancy (57, 58). Other proteins that were upregulated post-partum were the chemokines CCL8, CCL13 and CXCL5. CCL8 and CCL13 are both involved in the chemotaxis of monocytes and have been associated with MS (59). CCL8 has been implicated in microglia activation and was detected in inflammatory cells in MS lesions (59). CCL13 is also believed to be linked to the activation of oligodendrocytes and myelin destruction (60). Finally, CXCL5 is involved in the chemotaxis of neutrophils and has been found elevated in the plasma of MS patients in periods of formation of inflammatory lesions (61). Thus, speculatively, the upregulation of CCL8, CCL13, and CXCL5 post-partum, coinciding with increased disease activity, might be associated with disease-promoting mechanisms.

It is difficult to define the origin of the proteins measured in the plasma of pregnant women. However, it is likely that local production at the fetal-maternal interface, where most of the immune regulation is observed, contributes to the circulating cytokine levels. Indeed, most of the proteins that were upregulated in plasma during pregnancy are known to be produced at high levels by the placenta and by the uterine endometrium, named “decidua” during pregnancy. In the decidua, both infiltrating maternal immune cells and decidual stromal cells produce PD-L1, TGF-β, LIF, IL-10RB, CSF-1, and HGF (43, 62), and so do trophoblast cells in the placenta (42, 63). In particular, the syncytiotrophoblast, which is directly connected to the maternal inter-villous blood, produces high amounts of immune-modulatory proteins, both in free form and in microvesicles (64). The syncytiotrophoblast is also the main producer of pregnancy hormones during the latter part of pregnancy (65). The levels of the pregnancy hormones P4 and E2 did not differ between MS patients and HC and were closely correlated with the changes of the most regulated proteins during pregnancy. These are expected findings, given the dynamic hormonal pattern of gradual increase during and immediate drop after pregnancy, and since they are merely correlations, no conclusion can be drawn regarding a causal relationship. However, the immunoregulatory properties of P4 in particular (66–68) suggest that it is directly or indirectly involved in immune regulation during pregnancy by modulating the expression of the measured proteins.

Although the effect of pregnancy on MS and several other autoimmune diseases is long known (5, 69), surprisingly few studies have investigated it in the case of MS (9–14). There is only one previous report on cytokine profiling in MS patients during pregnancy, where seven proteins were measured in serum by ELISA (12). Although this study did include longitudinal samples, the only association found was the occurrence of relapses during pregnancy or post-partum when low levels of Activin-A and IL-10 were observed. In accordance with the timing of these changes, we found that focusing on the 3^rd^ trimester and post-partum as the time-points of major change in disease activity revealed many proteins that were differentially expressed in the 3^rd^ trimester and reversed post-partum, which was the case for all the top regulated proteins.

A strength of this study is the use of the highly sensitive PEA method, which gives the possibility to detect low abundant proteins with high specificity in various biological samples, including plasma (17). One potential limitation of our study is the small sample size. However, the samples were longitudinally collected so that inter-individual differences were overcome. Since our findings showed similar results in MS and HC, it was justified to compare our results with larger studies on healthy women (27, 28), which showed findings in line with ours. Due to the similarities between MS and HC, in additional analysis, we increased the statistical power by combining data from our MS patients and HC, and obtained very similar results, thus, the sample size was sufficient to show the robust and consistent changes that occurred during pregnancy. The longitudinal design and the collection of the pre-pregnancy sample in the MS group are important strengths, although it was not possible to obtain a pre-pregnancy sample in the HC group. Instead, we used an independent non-pregnant group, which yielded similar dynamic patterns as in the MS group. However, since there were relatively minor changes between pre-pregnancy and 1^st^ trimester samples, we used the 1^st^ trimester sample as the baseline for the detailed statistical analyses.

In summary, we here report plasma protein profiling in MS patients during pregnancy and identify consistent dynamic immunomodulatory changes that follow the pattern of maximal clinical improvement and infection severity peaking in the 3^rd^ trimester of pregnancy. Our results highlight proteins of potential mechanistic importance that could serve as potential biomarker candidates for MS amelioration (PD-L1, LIF-R, TGF-β1, and CCL28) or worsening (TRANCE, TWEAK, CCL8, CCL13, and CXCL5). Further functional studies would elucidate the mechanistic role of these proteins and additionally, clinical studies are needed to reveal their utility as biomarkers.

## Data Availability Statement

The raw data can be found in **Table S2** in the article/Supplementary Material and the R-code used to analyze the data is available in the github repository at https://github.com/sanhe374/MS_Pregnancy_proteomics.

## Ethics Statement

This study was reviewed and approved by the Regional Ethics review board in Linköping (2012/402-31), Sweden. The patients/participants provided their written informed consent to participate in this study.

## Author Contributions

SH, HC and MK handled blood samples. GPL, SH, and JH performed analysis of protein data together with MG and JE. TO, MK, MV, JM, JL, MS and MB recruited patients and controls and/or collected clinical data. SH, MS, TO, MK, IT, IK, JE, MJ and MV were involved in the overall design of the study. GPL was responsible for making the figures and drafting the manuscript with the support of SH and JE. All authors contributed to the article and approved the submitted version.

## Funding

The study was funded by the Swedish Foundation for Strategic Research (SB16-0011), the Swedish Research Council (2018-02776, 2021-03092), the Medical Research Council of Southeast Sweden (FORSS-315121), NEURO Sweden (F2018-0052), ALF grants, Region Östergötland and the Swedish Foundation for MS Research.

## Acknowledgments

The authors would like to acknowledge support of the Clinical biomarker facility at SciLifeLab Sweden for providing assistance in protein analyses. We would also especially like to acknowledge Gunn Johansson (Department of Neurology, Linköping University Hospital), Therese Pollack and Gunn Jönsson (Department of Neurology, Karolinska) and Henriette Ronnemo (Department of Neurology, Region Jönköping County) for help with all patient samples. To all the nurses and midwives at Familjecentrum, Region Kalmar County, in particular Carina Nilsson, for help with recruiting and taking samples from all healthy pregnant women. We further acknowledge the help of the flow cytometry unit at the Department of Laboratory Medicine, Region Jönköping County, for taking care of the patient samples recruited in Jönköping. Last but not least, we also thank Petra Cassel (Department of Clinical Immunology and Transfusion Medicine, Linköping University Hospital) for help with sample collection.

## Conflict of Interest

IT has served at advisory board for Pfizer Inc. JM has received honoraria for Advisory boards for Sanofi Genzyme and Merck and lecture honorarium from Merck. TO has received grant support from the Swedish Research Council, the Swedish Brain foundation, The Knut and Wallenberg foundation and Margaretha af Ugglas foundation and has received compensation for lectures/advisory boards, and unrestricted MS research grants from Biogen, Novartis, Sanofi and Merck. JE has received compensation for lectures from AbbVie, Biogen and Merck

The rest of the authors declare that the research was conducted in the absence of any commercial or financial relationships that could be construed as a potential conflict of interest.

The reviewer SS declared a past co-authorship with one of the authors JE to the handling editor.

## Publisher’s Note

All claims expressed in this article are solely those of the authors and do not necessarily represent those of their affiliated organizations, or those of the publisher, the editors and the reviewers. Any product that may be evaluated in this article, or claim that may be made by its manufacturer, is not guaranteed or endorsed by the publisher.

## Abbreviations

CNS, Central nervous system; DEPs, Differentially expressed proteins; E2, Estradiol; EAE, Experimental autoimmune encephalomyelitis; FDR, False discovery rate; HC, Healthy controls; LOD, Limit of detection; MS, Multiple sclerosis; P4, Progesterone; PCA, Principal component analysis; PEA, Proximity extension assay; Treg, Regulatory T cell.
