# Erratum: Plasma protein profiling reveals dynamic immunomodulatory changes in multiple sclerosis patients during pregnancy

**DOI:** 10.3389/fimmu.2024.1494595

**Published:** 2024-09-20

**Authors:** Frontiers Production Office

**Affiliations:** Frontiers Media SA, Lausanne, Switzerland

**Keywords:** multiple sclerosis, pregnancy, Olink proteomics, plasma, inflammation, cytokines, hormones

Due to a production error, the line “113/33 proteins, odds ratio (OR): 98.9, p=4.85e-09, Fisher’s exact test, Figure 4B” was incorrectly inserted.

A correction has been made to the section “**Results**”, sub-section “**Differentially expressed proteins during pregnancy in MS**”, paragraph three. The corrected text appears below:

“13/33 proteins, odds ratio (OR): 98.9, p=4.85e-09, Fisher’s exact test, Figure 4B)”

Due to the same error, the line “remains to be determined” was also incorrectly omitted.

A correction has been made to the section “**Discussion**”, paragraph two. The corrected text appears below:

“However, whether the circulating levels of IL-10RB and CSF-1 are associated with beneficial effects on CNS inflammation remains to be determined.”

The publisher apologizes for these mistakes.

The original version of this article has been updated.

