# Supplementary material for: Plasma protein profiling reveals dynamic immunomodulatory changes in multiple sclerosis patients during pregnancy
Source: Front Immunol. 2022 Jul 29;13:930947. doi: 10.3389/fimmu.2022.930947 (PMC9373039; doi:10.3389/fimmu.2022.930947)
Supplement: Supplementary file 1 [file DataSheet_1.docx]

Supplementary Material

**Plasma protein profiling reveals dynamic immunomodulatory changes in multiple sclerosis patients during pregnancy**

Georgia Papapavlou Lingehed^1†*^, Sandra Hellberg^2†^, Jesse Huang^3^, Mohsen Khademi^3^, Ingrid Kockum^3^, Hanna Carlsson^4^, Ivar Tjernberg^4^, Maria Svenvik^5^, Jonas Lind^6^, Marie Blomberg^7^, Magnus Vrethem^8^, Johan Mellergård^8^, Mika Gustafsson^2^, Maria C. Jenmalm^1^, Tomas Olsson^3^, Jan Ernerudh^9^

^1^Division of Inflammation and Infection, Department of Biomedical and Clinical Sciences, Linköping University, Linköping, Sweden

^2^Division of Bioinformatics, Department of Physics, Chemistry and Biology, Linköping University, Linköping, Sweden

^3^Neuroimmunology Unit, Department of Clinical Neuroscience, Center for Molecular Medicine, Karolinska University Hospital, Karolinska Institute, Stockholm, Sweden

^4^Department of Clinical Chemistry and Transfusion Medicine, Region Kalmar County, and Department of Biomedical and Clinical Sciences, Division of Inflammation and Infection, Linköping University, Linköping, Sweden

^5^Department of Obstetrics and Gynecology, Region Kalmar County, Kalmar, Sweden and Department of Biomedical and Clinical Sciences, Linköping University, Linköping, Sweden

^6^Section of Neurology, Department of Internal Medicine, County Hospital Ryhov, Jönköping, Sweden and Department of Biomedical and Clinical Sciences, Linköping University, Linköping, Sweden

^7^Department of Obstetrics and Gynecology in Linköping, and Department of Biomedical and Clinical Sciences, Linköping University, Linköping, Sweden

^8^Department of Neurology, and Department of Biomedical and Clinical Sciences, Linköping University, Linköping, Sweden

^9^Department of Clinical Immunology and Transfusion Medicine, and Department of Biomedical and Clinical Sciences, Linköping University, Linköping, Sweden

*** Correspondence:**Georgia Papapavlou Lingehed
[georgia.papapavlou.lingehed@liu.se](mailto:georgia.papapavlou.lingehed@liu.se)

^†^These authors have contributed equally to this work and share first authorship.

# Supplementary Figures


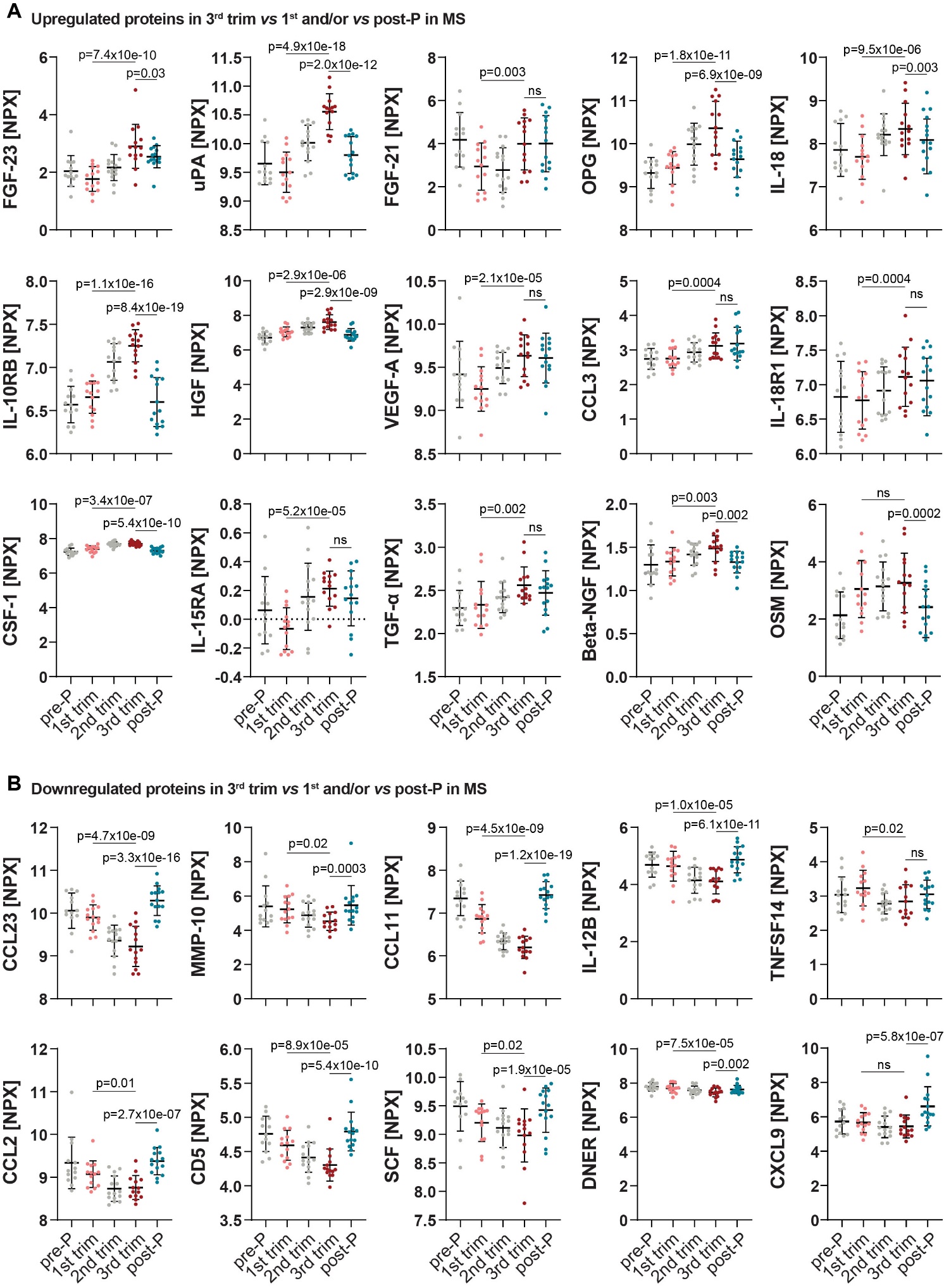

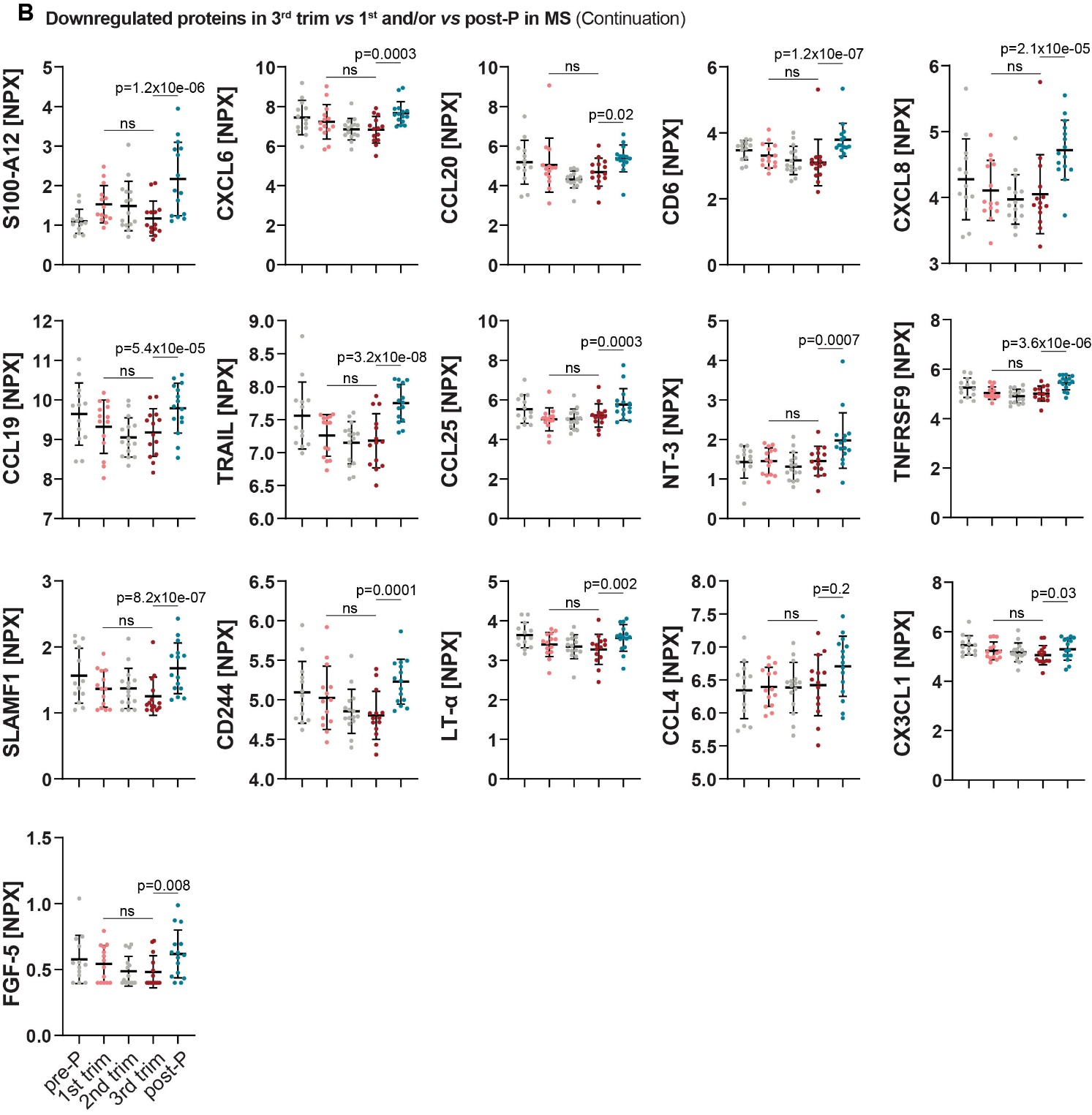


**Supplementary Figure S1.** Differentially expressed proteins in 3^rd^ trimester *versus* 1^st^ and/or *versus* post-partum in MS. The top five up- and down-regulated proteins are shown in **Figure 3**, and here (**A, B**) dot plots are shown of the additional differentially expressed proteins which were up- or down-regulated in the 3^rd^ trimester *versus* 1^st^ and/or *versus* post-partum in MS patients. To visualize the entire dynamic pattern, the time-points pre-pregnancy and 2^nd^ trimester are also shown, but in grey and without p-values. Protein levels are presented as NPX values on a log2 scale. All proteins had an FDR adjusted p-value < 0.05 (linear models and Benjamini-Hochberg correction, see **Table S3** for more details). Mean ± standard deviation is shown. MS; multiple sclerosis, NPX; normalized protein eXpression, pre-P; pre-pregnancy, post-P; post-partum, trim; trimester.


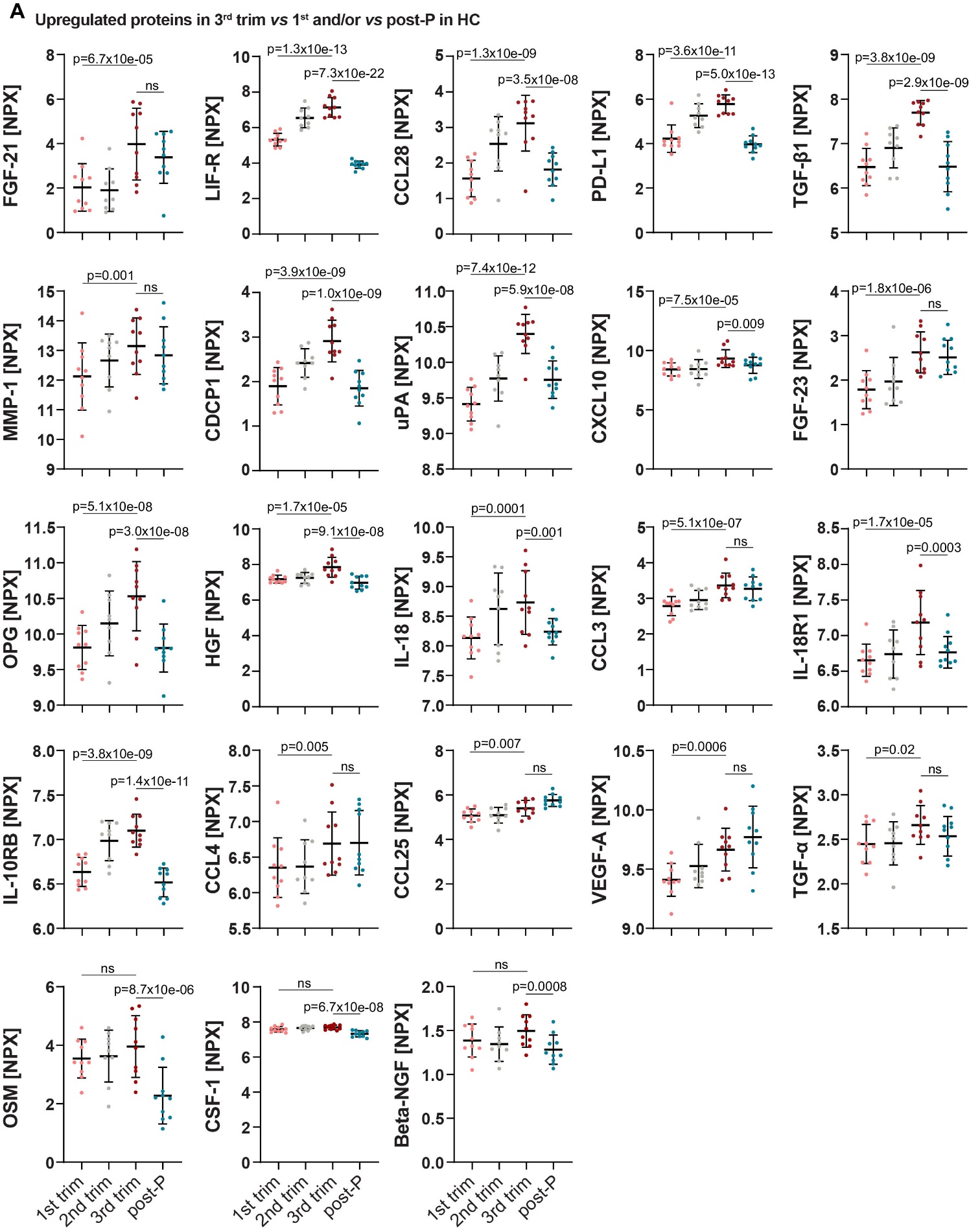

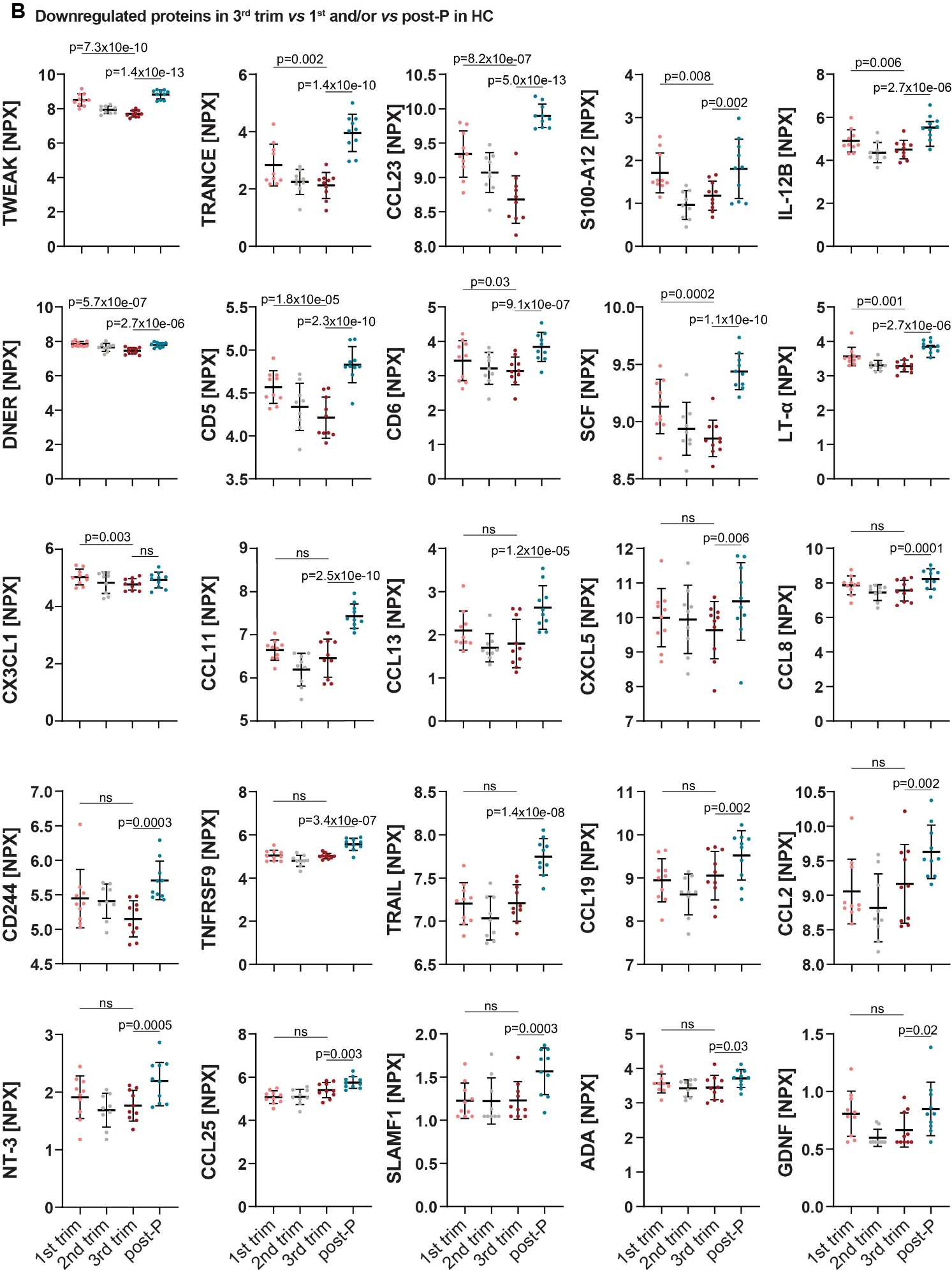
 **Supplementary Figure S2.** Differentially expressed proteins in 3^rd^ trimester *versus* 1^st^ and/or *versus* post-partum in HC. (**A, B**) Dot plots of the differentially expressed proteins which were up- or down-regulated in the 3^rd^ trimester *versus* 1^st^ and/or *versus* post-partum in healthy controls. To visualize the entire dynamic pattern, the 2^nd^ trimester time-point is also shown, but in grey and without p-values. Protein levels are presented as NPX values on a log2 scale. All proteins had an FDR adjusted p-value < 0.05 (linear models and Benjamini-Hochberg correction, see **Table S4** for more details). Mean ± standard deviation is shown. HC; healthy controls, NPX; normalized protein eXpression, post-P; post-partum, trim; trimester.


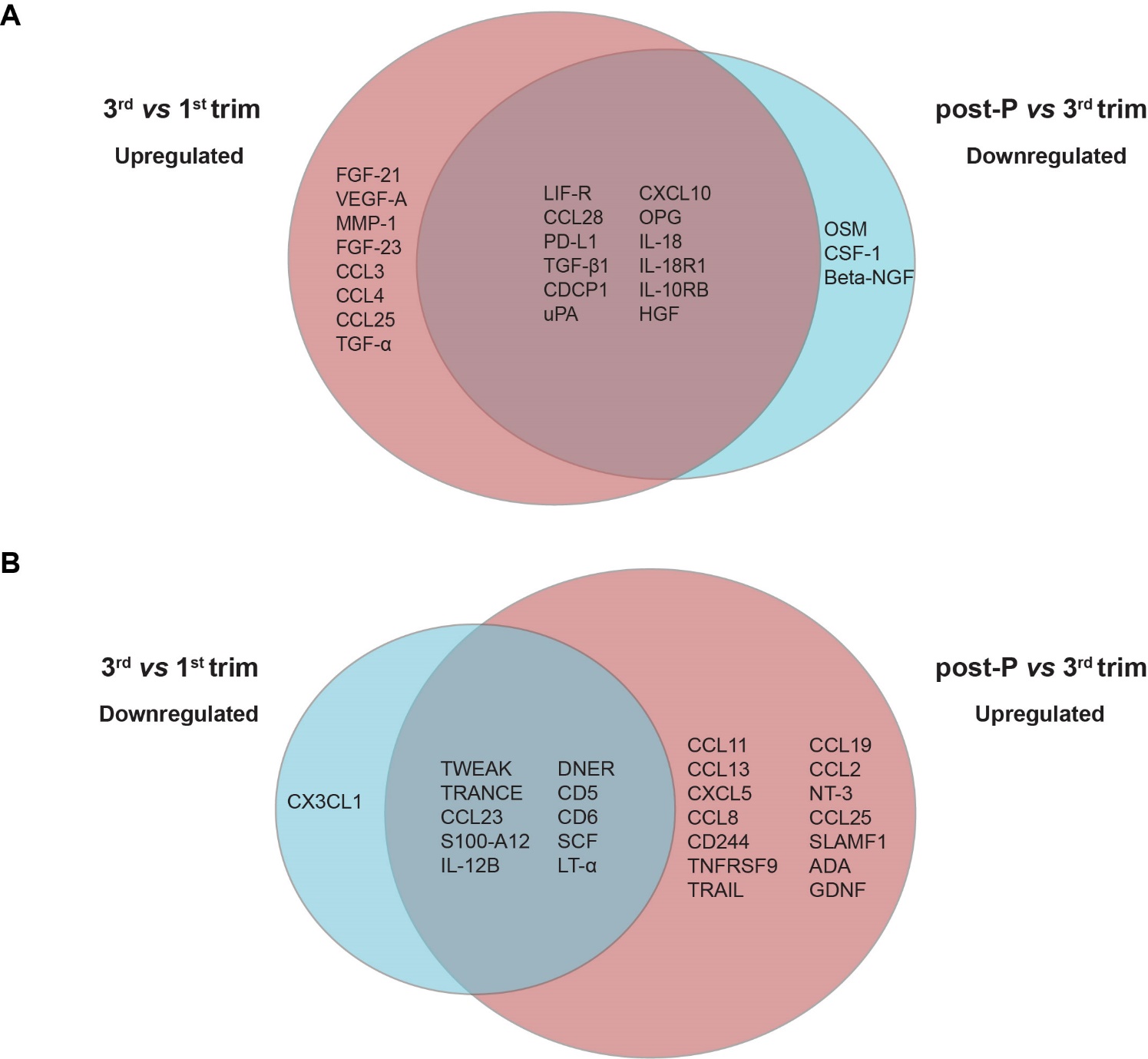


**Supplementary Figure S3.** Differentially expressed proteins in HC. **(A, B)** Venn diagrams depicting the common and unique differentially expressed proteins (DEPs) comparing up- and downregulated DEPs in 3^rd^ *versus* 1^st^ trimester and post-partum *versus* 3^rd^ in healthy controls. post-P; post-partum, trim; trimester.


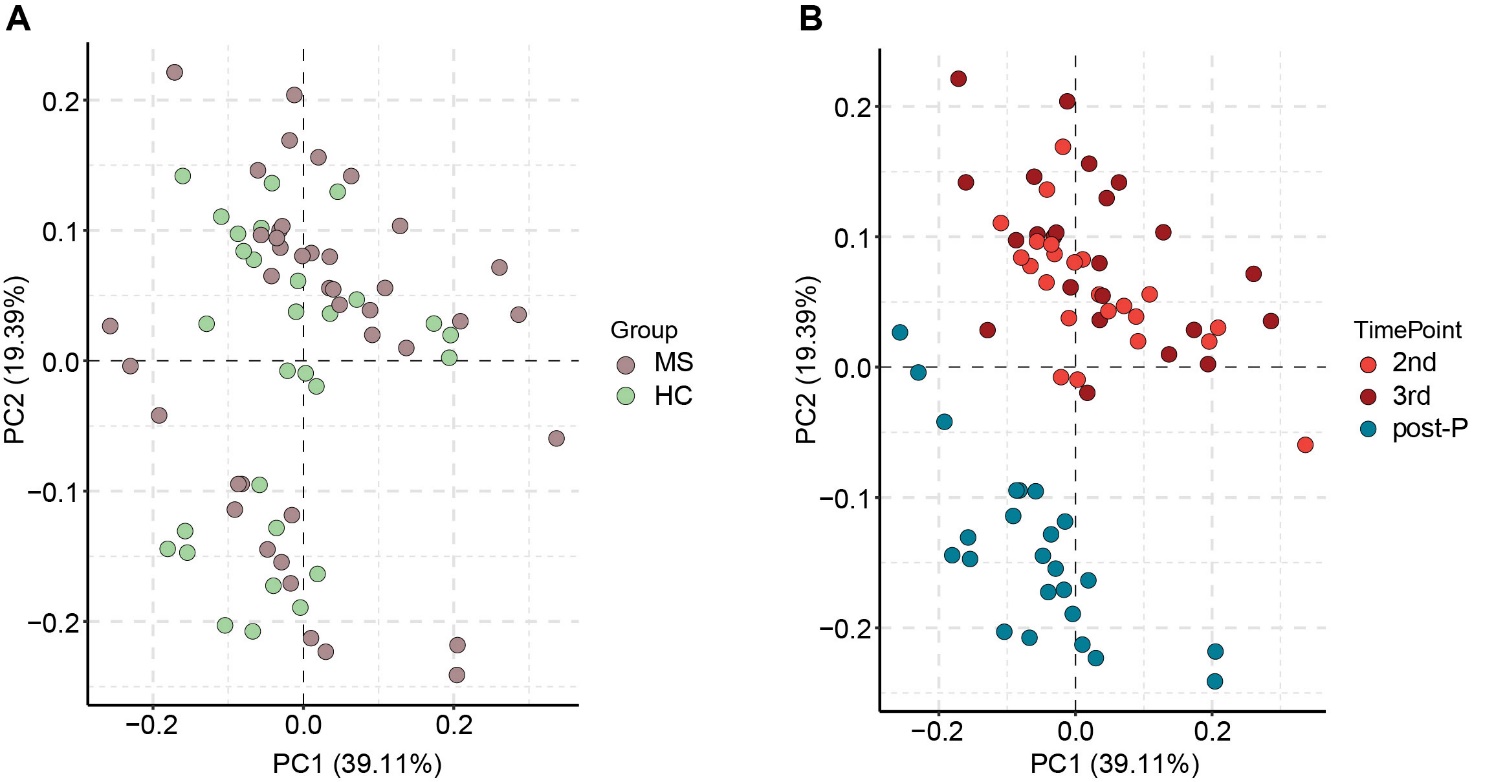


**Supplementary Figure S4.** Principal component analysis. **(A)** Principal component analysis (PCA) plots of all samples by group and time-point. Brown dots represent MS patients (n=14, individual 4 is excluded due to missing 1^st^ trimester sample) and green dots represent healthy controls (n=10). Light red dots represent 2^nd^ trimester samples, dark red 3^rd^ trimester and blue dots post-partum samples. HC; healthy controls, MS; multiple sclerosis, PC; principal component, post-P; post-partum.


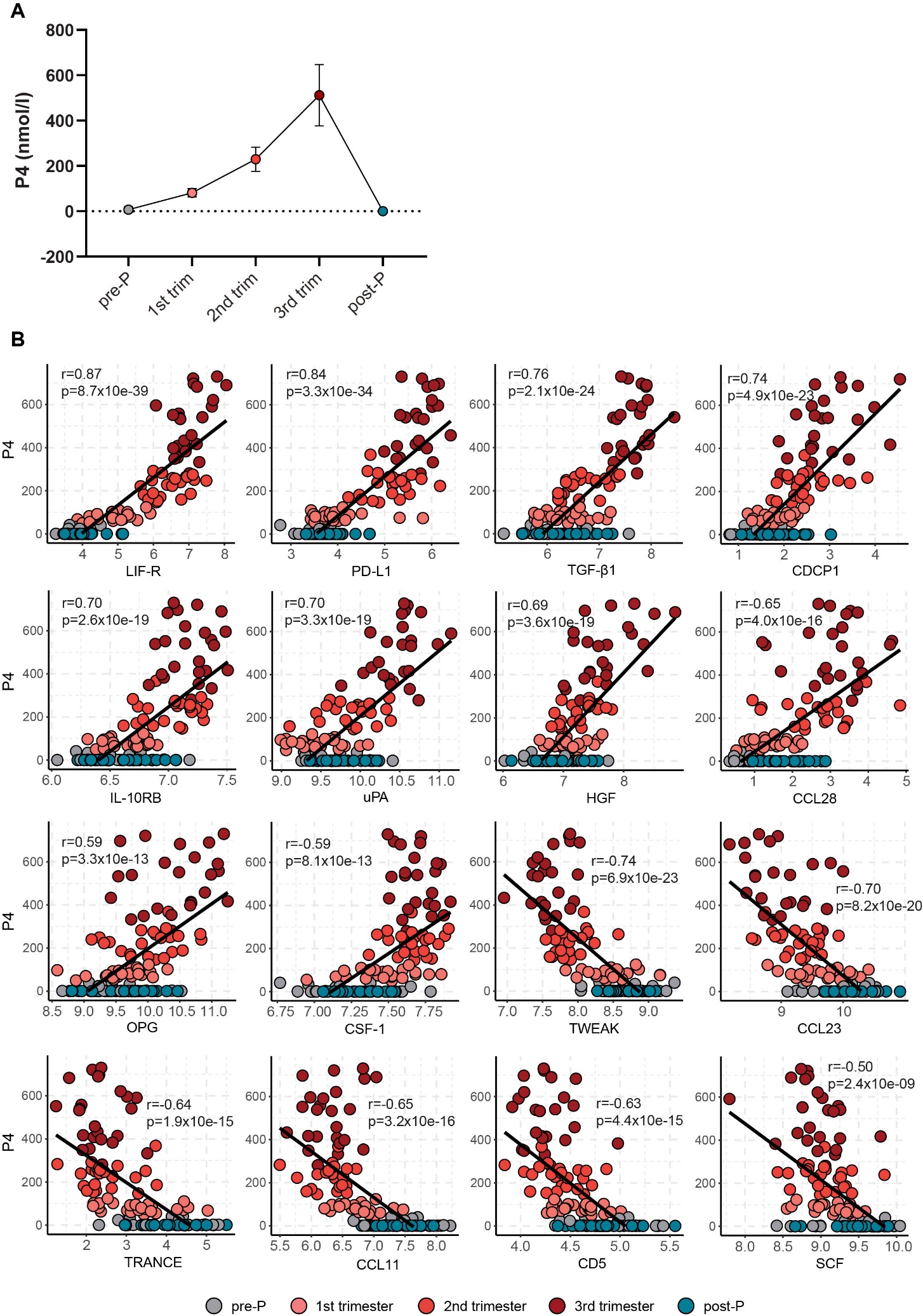


**Supplementary Figure S5.** Protein correlation with progesterone levels. **(A)** Progesterone (P4) levels in nmol/l in all plasma samples from MS patients and healthy controls (HC) before, during (1^st^, 2^nd^, and 3^rd^ trimesters) and after pregnancy. For the pre-pregnancy sample in the HC group, an independent non-pregnant group was included (n=14). The total number of samples per time-point is the following: pre-pregnancy (n=27), 1^st^, 2^nd^ and 3^rd^ trimesters (n=24), and post-partum (n=25). **(B)** Pearson correlations between P4 and protein levels. Proteins with correlation coefficient r > 0.5 in absolute value and a p-value < 0.05 were considered significant. Proteins are ordered according to descending r value, with positive correlations presented first, followed by negative correlations. P4: progesterone, pre-P; pre-pregnancy, post-partum, trim; trimester.


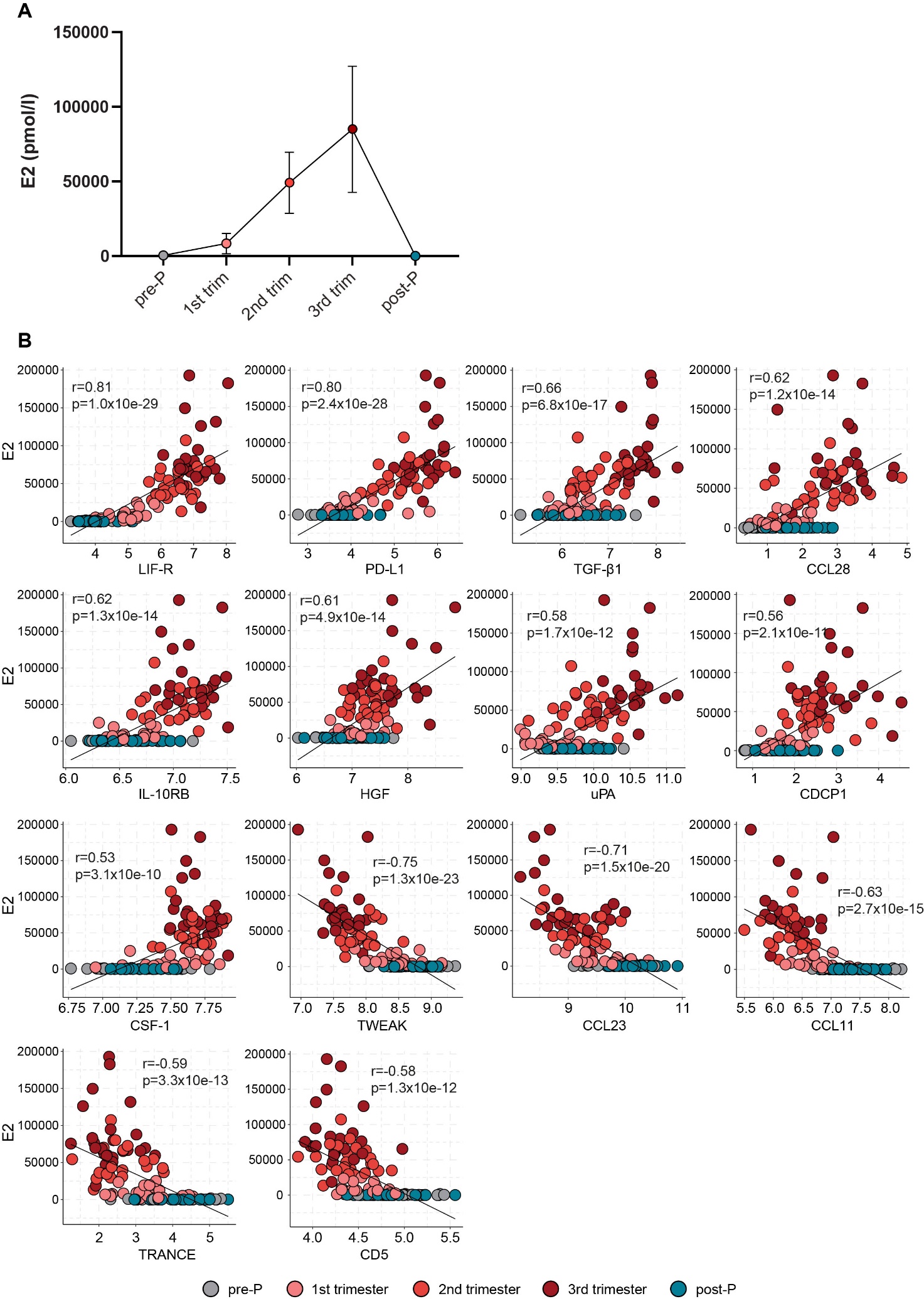


**Supplementary Figure S6.** Protein correlation with estradiol levels. **(A)** Estradiol (E2) levels in pmol/l in all plasma samples from MS patients and healthy controls (HC) before, during (1^st^, 2^nd^, and 3^rd^ trimesters) and after pregnancy. For the pre-pregnancy sample in the HC group, an independent non-pregnant group was included (n=14). The total number of samples per time-point is the following: pre-pregnancy (n=27), 1^st^, 2^nd^ and 3^rd^ trimesters (n=24), and post-partum (n=25). **(B)** Pearson correlations between E2 and protein levels. Proteins with correlation coefficient r > 0.5 in absolute value and a p-value < 0.05 were considered significant. Proteins are ordered according to descending r value, with positive correlations presented first, followed by negative correlations. E2; estradiol, pre-P; pre-pregnancy, post-partum, trim; trimester.

# Supplementary Tables

**Supplementary Table S1.** List of the 92 proteins analyzed in plasma samples from MS patients and controls with proximity extension assay using the Inflammation panel (protein name and UniProt ID available). The call rate (%) is stated, where 50% was the cut-off (values above 50% are marked green, and below are marked orange). Limit of detection (LOD) is given as a log2 transformed value.

**Supplementary Table S2.** Raw data of plasma mediators. Values of the 92 inflammation-related proteins measured by proximity extension assay technology and the pregnancy hormones progesterone (P4) and estradiol (E2) measured by electrochemiluminescence in plasma of multiple sclerosis patients and healthy controls. Protein levels are presented on a log2 scale, P4 is expressed in nmol/l and E2 in pmol/l. Individuals are coded with alphabet letters and timepoints with numbers; 0; pre-pregnancy, 1;1^st^ trimester, 2; 2^nd^ trimester, 3; 3^rd^ trimester and 4; post-partum.

**Supplementary Table S3.** Differentially expression analysis in MS patients. The results from the differentially expression analysis of plasma protein levels in multiple sclerosis (MS) patients are shown for all possible paired comparisons between the timepoints pre-pregnancy (pre-P), during pregnancy (1^st^, 2^nd^, and 3^rd^ trimesters) and post-partum (post-P).

**Supplementary Table S4.** Differentially expression analysis in HC. The results from the differentially expression analysis of plasma protein levels in healthy controls (HC) are shown for all possible paired comparisons between the timepoints during pregnancy (1^st^, 2^nd^ and 3^rd^ trimesters) and post-partum (post-P).
